# Exercise intervention for the management of chemotherapy-induced peripheral neuropathy: a systematic review and network meta-analysis

**DOI:** 10.3389/fneur.2024.1346099

**Published:** 2024-01-30

**Authors:** Natsuki Nakagawa, Sena Yamamoto, Akiko Hanai, Ayano Oiwa, Harue Arao

**Affiliations:** ^1^Department of Respiratory Medicine, The University of Tokyo Hospital, Tokyo, Japan; ^2^Division of Health Sciences, Osaka University Graduate School of Medicine, Osaka, Japan; ^3^Advanced Data Science Project, RIKEN, Yokohama, Japan; ^4^Division of Pain Clinic, Department of Anesthesiology, The Jikei University School of Medicine, Tokyo, Japan

**Keywords:** chemotherapy induced peripheral neuropathy, exercise, quality of life, cancer, systematic review & meta-analysis

## Abstract

**Purpose:**

Although exercise is recommended for cancer survivors with chemotherapy-induced peripheral neuropathy (CIPN), the effective types of exercise for preventing and treating CIPN remain unclear. This systematic review and network meta-analysis (NMA) aimed to evaluate the comparative effects of exercise on CIPN.

**Methods:**

We included relevant randomized controlled trials (RCTs) identified in a 2019 systematic review that evaluated the effects of exercise on CIPN and conducted an additional search for RCTs published until 2023. We evaluated the risk of bias for each RCT; the comparative effectiveness of exercise on patient-reported quality of life (QOL) through an NMA; and the effectiveness of exercise on QOL scores, patient-reported CIPN symptoms, and pain through additional meta-analyses.

**Results:**

Twelve studies (exercise, *n* = 540; control, *n* = 527) comparing 8 exercise interventions were included in the analysis. All studies were determined to have a high risk of bias. The meta-analyses showed significantly improved QOL [standard mean differences (SMD) 0.45; 95% confidence interval (CI) = 0.12 to 0.78] and CIPN symptoms (SMD 0.46; 95% CI = 0.11 to 0.82). No severe adverse events were reported. Pain tended to improve with exercise (SMD 0.84; 95% CI = −0.11 to 1.80). An NMA suggested that the interventions of a combination of balance and strength training showed a significant improvement in QOL scores compared to the control.

**Conclusion:**

Exercise interventions may be beneficial for improving QOL and CIPN symptoms. High-quality large clinical trials and data are needed to conclude that exercise is beneficial and safe.

## 1 Introduction

Approximately half of adult cancer patients experience numbness or tingling, symptoms collectively recognized as chemotherapy-induced peripheral neuropathy (CIPN) while receiving neurotoxic chemotherapy (1). CIPN diminishes over time after chemotherapy is completed, with a 30% prevalence at 6 months after completion of chemotherapy (2). In some patients, symptoms persist for several years (3). CIPN can result in dose reduction or discontinuation of cancer chemotherapy (4, 5). The intractable and chronic nature of CIPN can lead to functional and psychiatric impairment, including slower gait and a higher risk of falls compared to cancer patients without CIPN (6–8). CIPN can seriously adversely affect quality of life (QOL) (2). Recent clinical guidelines on CIPN assert that there is no method strongly recommended for its prevention or treatment (9, 10). They note that duloxetine is the only medication with sufficient evidence supporting its use in CIPN patients experiencing established pain (9, 10). Recent studies suggest that its effectiveness is comparable to that of a placebo (11).

Previous studies have reported that physical activity and exercise improve cardiovascular fitness, muscle strength, health-related QOL, depression, cachexia, and cancer-related fatigue (12–15). The guidelines of the American Society of Clinical Oncology (ASCO) for patients receiving active cancer treatment recommend regular exercise as supportive or palliative care (16).

Although exercise therapy for CIPN mitigation has not been established as part of any established guideline, several systematic reviews imply the potential efficacy of exercise for the prevention and treatment of symptoms related to CIPN (10, 11, 13, 17–19). In these studies, the dose, frequency, intensity, and duration of exercise programs varied (i.e., ranging from inactivity avoidance instructions to resistance training), and the outcome (e.g., specific sensorimotor functions, activities of daily living, QOL) also varied across the studies. According to the ASCO CIPN guidelines, exercise therapy is not currently recommended as a preventative or treatment approach for CIPN because of the low quality of the available evidence based on a systematic review of the literature published between January 2013 and August 2019 (10). The guidelines state that further robust research is necessary to determine the efficacy and potential risks of exercise therapy (10). In a systematic review conducted by Lin et al. (17), the overall mean effect size was estimated using standardized mean differences and a fixed-effect model, and significant improvement in the exercise cohort compared to the control group was reported (mean difference: 0.5319; 95% confidence interval: 0.2295 to 0.8344; *Z* = 3.45). The study suggested the need for further investigation of different exercise protocols and intervention intensities with larger sample sizes and more specific outcome measures (17). Since the publication of these systematic reviews, additional clinical trials of exercise on CIPN have been published.

The primary research question was “How effective are exercise interventions for people with CIPN?.” We aimed to update the systematic reviews of randomized controlled trials (RCTs) included in the ASCO clinical guidelines by incorporating additional recently published studies evaluating the effect of exercise on the mitigation of CIPN symptoms with an additional focus on the type of exercise program. Since CIPN symptoms and their incidence rate vary by assessment measurements (20), a consensus has been reached that clinical trials investigating CIPN should include an assessment of QOL as an indicator that best reflects the patient’s life state considering various complex symptoms (21). We conducted a meta-analysis with QOL as a primary outcome, and specific outcomes of CIPN symptoms were also analyzed to capture changes in CIPN symptoms associated with exercise interventions. To focus on the details and diversity of exercise intervention programs, we conducted a network meta-analysis (NMA) to determine which exercise interventions were most effective in improving QOL.

## 2 Method

### 2.1 Study design and protocol

We conducted a systematic review and meta-analysis, which followed the guidelines of the Preferred Reporting Items for Systematic Reviews and Meta-Analyses (PRISMA) statement (22). The review protocol was prospectively registered with the University Hospital Medical Information Network Clinical Trials Registry (UMIN000045558). Ethical approval and informed consent were waived, considering the nature of the study. This study was conducted as a part of the Japanese clinical practice guidelines on CIPN and was based on a clinical guideline previously published by ASCO (10). In the scope of guideline development, the choice was made to include all randomized controlled trials comprising the ASCO systematic review as part of the current analysis (23, 24).

### 2.2 Literature search and data sources

In accordance with the guideline committee policy, a systematic literature search targeted studies published after September 2019 because a previous systematic literature search had been conducted in earlier clinical guidelines published by ASCO (10). A systematic literature search was performed in PubMed on April 20, 2023, to identify RCTs published between September 1, 2019, and April 20, 2023, that evaluated the effects of exercise on CIPN and included more than five patients in the exercise group. Interventions specialized only for the upper or lower extremities were excluded. Supplementary Table S3 presents the search terms used in the publication search strategy. The literature search in PubMed was restricted to articles published in peer-reviewed journals and written in English. In addition to the systematic literature search, we evaluated studies manually obtained primarily by reviewing previously reported clinical guidelines and systematic reviews (10, 11, 13, 17, 25). Two authors independently screened each study for eligibility, and disagreements among reviewers were resolved through discussion.

### 2.3 Data extraction

Two reviewers (SY and NN) independently collected the following information from each included study using a predefined data extraction form: (1) general information about the study (author, year of publication, country, and study design); (2) sample size; (3) patient characteristics (age, sex, cancer type, anticancer drugs that could induce peripheral neuropathy, and the presence of baseline CIPN); (4) exercise type, intensity, duration and frequency; (5) examined outcomes, including CIPN; (6) timing of assessment of outcomes; (7) adverse effects related to the intervention; and (8) enrollment rate, completion rate of the study, and adherence to the intervention. The outcome measures were grouped into six categories referring to a MASCC book (25) as follows: (1) QOL (primary outcome); (2) patient-reported CIPN; (3) pain; (4) clinical assessments of CIPN signs; (5) balance measures; and (6) physical functional assessments. The enrollment rate was defined as the proportion of screened persons who were randomly assigned. The completion rate was defined as the proportion of randomly assigned persons for whom a final analysis was performed. The definition of adherence to exercise intervention followed the individual studies. Discrepancies between the reviewers were resolved through discussion or by a third reviewer (HA).

### 2.4 Quality assessment

Two reviewers (SY and NN) independently assessed the risk of bias using version 2 of the Cochrane risk of bias tool for randomized trials (RoB 2) (26). All disagreements among reviewers were resolved through discussion. Risk of bias assessments were summarized in a traffic-light plot using Risk of bias VISualization (robvis) (27). We could not assess the risk of bias, such as heterogeneity among the studies or publication bias, across the studies because many studies included outcomes of different types of measurements and did not specify a primary outcome variable. A funnel plot was used to check for the presence of publication bias.

### 2.5 Data synthesis and meta-analyses

We conducted a meta-analysis on outcome data regarding QOL (primary outcome), patient-reported CIPN symptoms, pain, and collected postintervention or a time point similar to postintervention. We included the studies that reported the data able to calculate the mean and standard deviation at each point. The standardized mean difference (SMD) was calculated due to different scales used across the study outcome measures. Confidence intervals were calculated as a measure of precision for the SMD estimates. If data were unavailable, we contacted the authors and used all data provided by the authors after contact. If a trial was conducted with multiple parallel arms including different types of exercise, we compared the outcomes by combining the arms against a control. The overall mean effect sizes were estimated using random-effects models. Heterogeneity was evaluated with chi-squared tests and *I*-square tests. We used Review Manager (RevMan, Version 5.4., The Cochrane Collaboration, 2020) for the meta-analysis.

### 2.6 Network meta-analysis of exercise intervention effectiveness and QOL

NMA is a technique for comparing some interventions in a single analysis by combining both direct and indirect reports including clinical trials (26). NMA was conducted to assess the comparative effectiveness of the different exercise interventions in each trial, and the results represent estimates of the relative effects between any given intervention pair in the NMA of QOL. The NMA was conducted using the “BUGSnet” package in R and was based on Bayesian methods (28). We specified 100,000 iterations with 10,000 burn-ins and 10,000 adaptations based on random- and fixed-effects models and compared their accuracies; we employed the model with the lowest deviance information criterion (DIC) and used Bayesian Markov chain Monte Carlo modeling. The treatments were ranked according to cumulative rank curve under surface (SUCRA) probabilities.

## 3 Results

### 3.1 Overview of the included literature and a flow diagram

**Figure 1:**
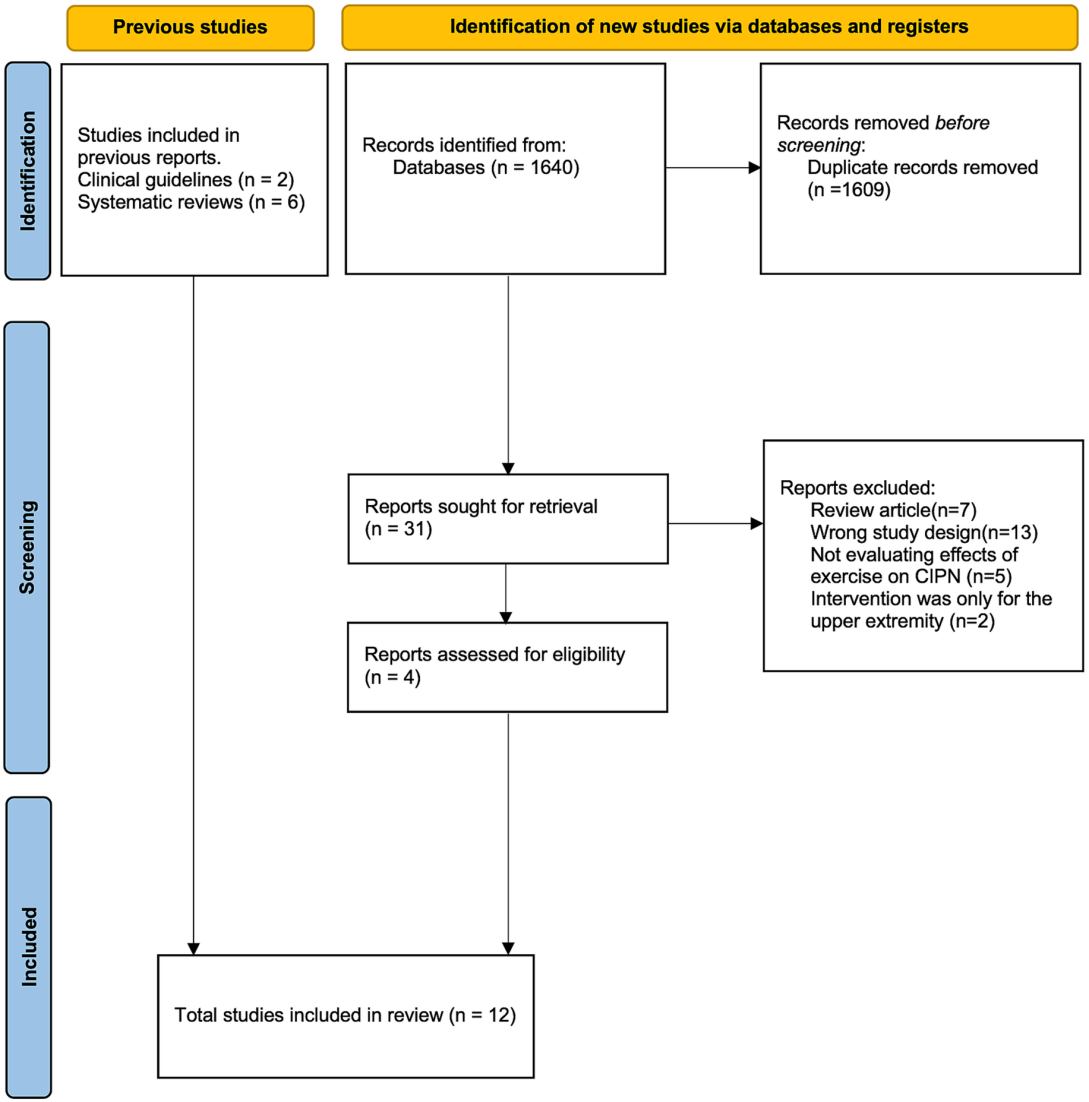
PRISMA flow diagram.

A total of 1,640 studies that were identified for screening after applying the search terms described in Supplementary Table S3 were screened, and 4 studies (24, 29–31) were included in the final analysis. In addition, the 2 studies (23, 32) included in the update to the ASCO guideline (10) and 6 additional studies (33–38) referred to in previous systematic reviews (11, 13, 17, 25) were included. In total, 12 randomized controlled trials were included in the qualitative synthesis (Figure 1).

Details on these studies, such as patient disease diagnosis, exercise intervention, and outcome measures, are provided in Supplementary Table S1. Two-arm (exercise vs. control) study designs were used in 8 studies (23, 24, 31–36); 3-arm designs were used in 2 studies (29, 30); and 4-arm designs were used in 2 studies (37, 38). The control conditions included usual care, education only, and different types of nonpharmacological interventions (Reiki, meditation, cold application). All the included studies aimed to assess the efficacy of exercise on CIPN. Intention-to-treat analysis was conducted in 5 studies (23, 24, 30, 31, 38), and per-protocol analysis was conducted in 2 studies (29, 33). In the remaining 5 studies (32, 34–37), it was unclear which analysis was conducted or how missing data were treated.

### 3.2 Risk of bias assessment

The risk of bias was assessed by funnel plots (Supplementary Figure S1), which were asymmetric and biased toward positive outcomes, and by the RoB 2 tool (Supplementary Figure S2). For randomization, 5 studies (24, 30, 32, 35, 36) were identified as having a high risk or some concerns for risk of bias, as they did not report detailed information about randomization and/or concealment, and one study clearly described that randomization was not masked (37). For deviation, all 12 studies were evaluated to have a high risk of bias. For missing outcome data, 11 studies were identified as high risk or with some concerns for risk of bias due to participants dropping out during the study (23, 24, 29–37). For measurement of the outcomes, 10 out of 12 studies were evaluated to have a high risk of bias because outcome assessors were often involved in the studies and were aware of the intervention (23, 24, 29, 30, 32–34, 36–38). Finally, for selection in the reported results, 3 studies were evaluated to have a high risk of bias because of discrepancies in the description between the methodology and reported results (24, 35, 38).

### 3.3 Study and patient characteristics

The sample sizes of the included studies ranged from 22 to 456 patients. In 6 studies (23, 24, 30, 34, 36, 37), the presence of CIPN before randomization was the eligibility criterion for the respective studies, while in 1 study (29), the absence of CIPN was the eligibility criterion. Patients with breast and gastrointestinal cancers formed the largest population. In all included studies, neurotoxic drugs were cytotoxic agents, and patients with CIPN caused by immune checkpoint inhibitors were excluded. Eight of the 12 studies dealt without separating the type of causative cytotoxic agents (platinum, taxane, vinca alkaloids).

### 3.4 Intervention details

The types of exercise interventions consisted of balance training (9 studies) (23, 24, 29, 30, 33–36, 38), strength training (7 studies) (23, 24, 29, 30, 32, 33, 35), aerobic training (4 studies) (23, 31–33), and yoga (37). The balance training intervention involved performing a task while maintaining an upright posture on an unstable platform (23, 24, 29, 30, 33–36, 38). Seven studies used a combination of different types of exercises (23, 24, 29, 30, 32, 33, 35). In 4 studies (24, 30, 32, 36), the intervention was conducted at home, largely unsupervised, with participants self-reporting their adherence. In 5 other studies (23, 33, 34, 37, 38), the intervention was conducted in the clinic, hospital or sports center, mostly under basic supervision, with an objective assessment of adherence. In one study, participants could choose the place where they would exercise (29). The length of the interventions ranged from 4 to 36 weeks in 11 studies (23, 24, 30–38). In 1 study (29), the intervention continued during chemotherapy, and the intervention duration was not decided beforehand. As shown in Supplementary Table S1, the frequency of exercise differed between the studies, ranging from 2 times a week to daily. Likewise, the length of time in which participants were engaged in exercise varied in each study. Most exercise interventions required less than an hour for each session. The intensity of aerobic exercise was adjusted based on the participants’ heart rate in all 3 studies in which the intervention included aerobic exercise (23, 32, 33). The intensity was adjusted based on individual capacity (23, 29, 30, 32, 33, 35) in 6 of the 7 studies in which the intervention included strength training. For other types of intervention, the intensity was maintained at a fixed level.

### 3.5 Outcome measurement tools

The primary outcome measures of each study are described in Supplementary Table S1. Patient-reported CIPN was the most frequent outcome measured (9 studies) (23, 24, 29–32, 34, 37, 38), and QOL was assessed in 8 studies (23, 24, 29, 31, 33, 35, 37, 38). Two studies assessed QOL using the European Organization for Research and Treatment of Cancer core quality of life questionnaire (EORTC-QLQ-C30) (39), and one study assessed QOL using FACT-Taxane (40). The other studies (23, 29, 35, 37, 38) did not detect significant differences in QOL and were assessed with the EORTC-QLQ-C30 or the Functional Assessment of Cancer Therapy-General (FACT-G) questionnaire (41). Three studies (23, 37, 38) assessed patient-reported CIPN symptoms using the Functional Assessment of Cancer Therapy/Gynecologic Oncology Group Neurotoxicity (FACT-GOG-NTX) questionnaire (42). Only one study evaluated the chemotherapy completion rate (29), but none of the studies assessed outcomes related to chemotherapy, including chemotherapy dose received or survival (Supplementary Table S2).

### 3.6 Effectiveness of exercise on patient-reported outcomes of QOL

**Figure 2:**
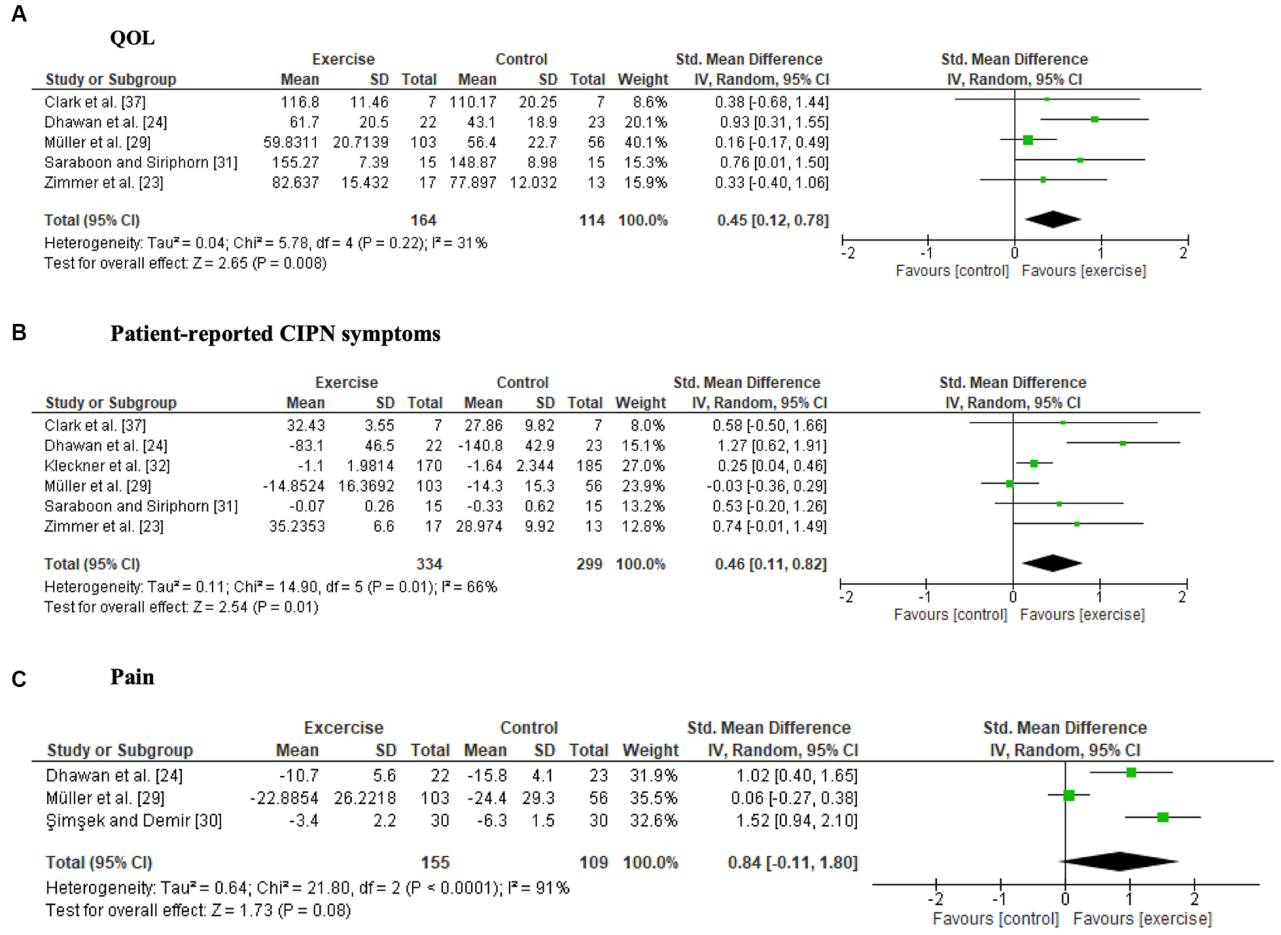
Forest plots of the effects of exercise on patient-reported outcomes. **(A)** Quality of life (QOL), **(B)** patient-reported CIPN symptoms, **(C)** pain.

Two studies (24, 33) reported a significant intervention effect on QOL. The synthesized data from 5 studies (23, 24, 29, 31, 37) revealed that exercise significantly improved QOL (SMD, 0.45; 95% CI = 0.12 to 0.78, *Z* = 2.65, *p* = 0.008, *I*^2^ = 31%) (Figure 2A).

### 3.7 Effectiveness of exercise for patient-reported CIPN symptoms

Four studies (23, 24, 30, 32) reported significant intergroup differences in patient-reported symptoms related to CIPN, whereas four studies (29, 31, 34, 38) did not detect significant differences. One study compared pre- and postintervention data and reported that neuropathic symptoms significantly worsened only in the control group (37). The six studies (23, 24, 29, 31, 32, 37) included in the meta-analysis showed a significant intervention effect on patient-reported CIPN symptoms (SMD, 0.46; 95% CI = 0.11 to 0.82, *Z* = 2.54, *p* = 0.01, *I*^2^ = 66%) (Figure 2B). Fear of falling was not included as a patient-reported CIPN symptom in the meta-analysis because of high heterogeneity (29, 34).

### 3.8 Adverse events related to exercise

Nine studies (23, 24, 29, 32–36, 38) reported adverse events; only Müller et al. (29) reported adverse events related to the exercise programs, in which pain, fatigue and dizziness occurred in 21%–25% of patients in the exercise group (29).

Both studies where pain was measured using pain scales (24, 38) reported a significant reduction in pain in the exercise group compared to the control group. There were no significant differences in pain scores on the Chemotherapy-Induced Peripheral Neuropathy Assessment Tool (CIPNAT) (30) or the EORTC-QLQ-C30 (29, 33, 38). A forest plot showed no significant difference between the exercise groups and control groups, but a trend towards a favorable effect of exercise was observed (SMD, 0.84; 95% CI = −0.11 to 1.80, *Z* = 1.73, *p* = 0.08, *I*^2^ = 91%) (Figure 2C).

### 3.9 Clinical assessments of CIPN post-exercise intervention

Clinical assessments of CIPN symptoms were significantly improved by exercise in 2 out of 3 studies (31, 33, 38). The beneficial effects of the intervention included improvements in peripheral deep sensitivity (33, 38) and Achilles tendon and patellar tendon reflexes (38). Significant intervention effects on balance ability were observed in all relevant studies (23, 29, 33–36). Outcomes related to balance involved multiple measures and a wide range of signs. Mixed results were observed for physical function. Four studies reported benefits to muscle strength (23, 29, 35) and SPPB (31), while three studies did not show beneficial effects (33, 34, 36).

### 3.10 Enrollment, completion rate and adherence

Patient enrollment rates were described in 7 studies (24, 29–31, 34, 35, 37) and ranged from 25% to 100% (Supplementary Table S4). Two studies (24, 35) described the reasons for the decrease in the enrollment rate, including lack of interest, personal reasons, and unfulfilled eligibility criteria. Completion rates were described in all studies and ranged from 64% to 100%. Six studies (23, 32, 34, 36–38) described the reasons for the decrease in the completion rate, including death, disease progression, and physical, psychological, and personal reasons. Adherence to the interventions was described in 8 studies (23, 24, 29, 31–33, 36, 38) and ranged from 49 to 98%. Two studies (29, 36) described the details of the reasons for the decrease in adherence, including side effects of anticancer treatment, time constraints and motivation.

### 3.11 Network meta-analysis of exercise interventions on QOL

**Figure 3:**
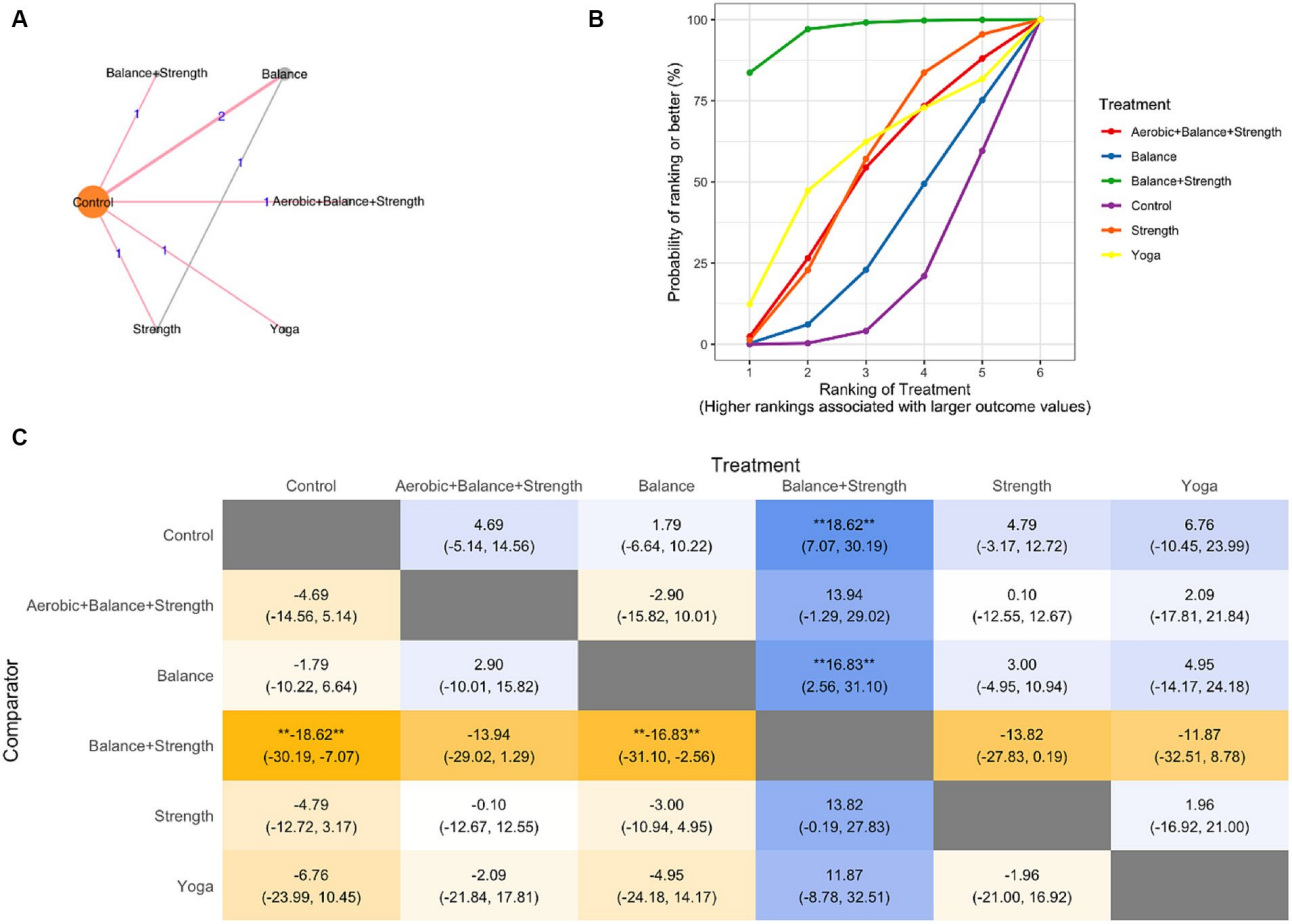
Network meta-analysis **(A)** network graph, **(B)** SUCRA plot, **(C)** League Table Heatmap. The values in each cell show the relative treatment effect and 95% credible intervals of the treatment on the top, compared to the treatment on the left. The double asterisks: statistical significance.

NMA was conducted to compare the effectiveness of each exercise intervention on QOL and included each trial arm for which a QOL index was available (23, 24, 29, 37) (Figure 3A network graph). The fixed-effects model was selected for this data set.

In the SUCRA plot (Figure 3B), the curve associated with the combination of treatment balance and strength training had the highest probability of being ranked first among treatments, suggesting that it is most likely to result in improved QOL scores. The model also generates effect estimates. The difference between balance and strength training and the other treatments was larger than that of the control and balance training (Figure 3C).

## 4 Discussion

Our analysis with updated trials indicated that exercise positively affected QOL, consistent with previous systematic reviews that showed that exercise resulted in an improvement in QOL scores (SMD: 14.62, 95% CI, 6.03–3.20) (19, 43). A similar meta-analysis of different studies on the severity of CIPN and peripheral deep sensitivity showed positive results, as in our research on QOL (44). Cancer survivors should keep active lifestyle (15), which requires motivation and appropriate exercise program selection (45–48). In our search, this is the first NMA study comparing different types of exercise interventions for CIPN, but the diversity of trials and outcomes could include bias. An RCT directly comparing balance training, strength training, and stretching resulted in different outcomes that showed efficacy (49); an NMA that suggested the efficacy of non-pharmacological interventions for CIPN also showed bias due to variability in trial setting (50).

There are three limitations of this research. First, most studies included are small and exploratory, with potential biases leading to positive results. Metrics used in the meta-analysis are not perfect, and the funnel plot suggests a selection bias. Factors like individual differences in exercise performance, unblinded interventions, and possible survival bias in patients adhering to protocols may contribute to this bias. Second, many studies did not identify the onset of CIPN and the timing of the intervention, making it unclear whether the purpose of the intervention was to prevent or treat CIPN. It was also difficult to identify the site of onset of CIPN, such as the fingertips only. Third, the studies in our review did not differentiate CIPN caused by various anticancer drugs, which is crucial as different drugs can cause distinct pathophysiologies. In diagnosing peripheral neuropathy, electromyography and nerve conduction velocity discern nerve fiber types (51). Nerve conduction studies (NCS) are suitable for detecting large fiber neuropathy but are often normal in small fiber neuropathy (SFN) and CIPN (52, 53). SFN can be diagnosed by skin biopsy in addition to symptom assessment (53–55). Definitive diagnostic methods for CIPN have not been established due to diverse mechanisms and inconsistency between subjective symptoms and neurophysiological testing, and invasive skin biopsy has limited its use in oncology (10, 52, 53, 55). Although the mechanism and symptoms of CIPN vary by anticancer drug, CIPN is characterized mainly by sensory nerve fiber damage (1, 9, 10). Complex neuroimmune interactions in the skin also affect CIPN and some types of drugs (bortezomib, thalidomide, and vincristine) can cause SFN symptoms, such as pain, temperature, and autonomic functions (9, 52–55). The studies in this SR do not adequately describe the neurological examination results or the specific effects of anticancer drugs on nerves. Relating to the exercise interventions proposed by some studies, it may be similar to the rehabilitative effects of exercise on diabetic peripheral neuropathy and small fiber damage (56), the actual mechanism of action of exercise on CIPN is unclear.

## 5 Conclusion

Our results suggest that exercise interventions may be beneficial for improving general quality of life and CIPN symptoms. High-quality clinical trials, including a larger number of subjects and data, are needed to conclude that exercise is beneficial and safe for the prevention and improvement of CIPN in many cancer patients.

## Data availability statement

The original contributions presented in the study are included in the article/Supplementary material, further inquiries can be directed to the corresponding author.

## Ethics statement

Ethical approval was not required for the study involving humans in accordance with the local legislation and institutional requirements. Written informed consent to participate in this study was not required from the participants or the participants’ legal guardians/next of kin in accordance with the national legislation and the institutional requirements.

## Author contributions

NN: Conceptualization, Data curation, Formal analysis, Investigation, Methodology, Project administration, Supervision, Validation, Writing – original draft, Writing – review & editing. SY: Conceptualization, Data curation, Formal analysis, Investigation, Methodology, Project administration, Resources, Software, Validation, Visualization, Writing – original draft, Writing – review & editing. AH: Formal analysis, Funding acquisition, Investigation, Project administration, Resources, Software, Supervision, Validation, Visualization, Writing – original draft, Writing – review & editing. AO: Conceptualization, Data curation, Methodology, Writing – original draft, Writing – review & editing. HA: Conceptualization, Data curation, Methodology, Supervision, Writing – review & editing.

## References

[1] Seretny M Currie GL Sena ES Ramnarine S Grant R MacLeod MR. Incidence, prevalence, and predictors of chemotherapy-induced peripheral neuropathy: a systematic review and meta-analysis. Pain. (2014) 155:2461–70. doi: 10.1016/j.pain.2014.09.020, PMID: 25261162

[2] Beijers A Mols F Dercksen W Driessen C Vreugdenhil G. Chemotherapy-induced peripheral neuropathy and impact on quality of life 6 months after treatment with chemotherapy. J Community Support Oncol. (2014) 12:401–6. doi: 10.12788/jcso.0086, PMID: 25856013

[3] Kneis S Wehrle A Müller J Maurer C Ihorst G Gollhofer A. It’s never too late—balance and endurance training improves functional performance, quality of life, and alleviates neuropathic symptoms in cancer survivors suffering from chemotherapy-induced peripheral neuropathy: results of a randomized controlled trial. BMC Cancer. (2019) 19:414. doi: 10.1186/s12885-019-5522-7 31046719 PMC6498676

[4] DiBonaventura MD Copher R Basurto E Faria C Lorenzo R. Patient preferences and treatment adherence among women diagnosed with metastatic breast cancer. Am Health Drug Benefits. (2014) 7:386–96. 25525495 PMC4268769

[5] Lyman GH. Impact of chemotherapy dose intensity on cancer patient outcomes. J Natl Compr Canc Netw. (2009) 7:99–108. doi: 10.6004/jnccn.2009.0009 19176210

[6] Winters-Stone KM Horak F Jacobs PG Trubowitz P Dieckmann NF Stoyles S. Falls, functioning, and disability among women with persistent symptoms of chemotherapy-induced peripheral neuropathy. J Clin Oncol. (2017) 35:2604–12. doi: 10.1200/JCO.2016.71.3552, PMID: 28586243 PMC5549452

[7] Shah A Hoffman EM Mauermann ML Loprinzi CL Windebank AJ Klein CJ. Incidence and disease burden of chemotherapy-induced peripheral neuropathy in a population-based cohort. J Neurol Neurosurg Psychiatry. (2018) 89:636–41. doi: 10.1136/jnnp-2017-317215, PMID: 29439162 PMC5970026

[8] Kolb NA Smith AG Singleton JR Beck SL Stoddard GJ Brown S. The association of chemotherapy-induced peripheral neuropathy symptoms and the risk of falling. JAMA Neurol. (2016) 73:860–6. doi: 10.1001/jamaneurol.2016.0383, PMID: 27183099 PMC6715416

[9] Jordan B Margulies A Cardoso F Cavaletti G Haugnes HS Jahn P. Systemic anticancer therapy-induced peripheral and central neurotoxicity: ESMO-EONS-EANO clinical practice guidelines for diagnosis, prevention, treatment and follow-up. Ann Oncol. (2020) 31:1306–19. doi: 10.1016/j.annonc.2020.07.003 32739407

[10] Loprinzi CL Lacchetti C Bleeker J Cavaletti G Chauhan C Hertz DL. Prevention and management of chemotherapy-induced peripheral neuropathy in survivors of adult cancers: ASCO guideline update. J Clin Oncol. (2020) 38:3325–48. doi: 10.1200/JCO.20.01399, PMID: 32663120

[11] Chow R Novosel M So OW Bellampalli S Xiang J Boldt G. Duloxetine for prevention and treatment of chemotherapy-induced peripheral neuropathy (CIPN): systematic review and meta-analysis. BMJ Support Palliat Care. (2023) 13:27–34. doi: 10.1136/spcare-2022-003815, PMID: 36194493

[12] Hong Y Wu C Wu B. Effects of resistance exercise on symptoms, physical function, and quality of life in gastrointestinal cancer patients undergoing chemotherapy. Integr Cancer Ther. (2020) 13:523–36. doi: 10.1007/s11764-019-00772-y PMC7493268 32909468

[13] Duregon F Vendramin B Bullo V Gobbo S Cugusi L Di Blasio A. Effects of exercise on cancer patients suffering chemotherapy-induced peripheral neuropathy undergoing treatment: a systematic review. Crit Rev Oncol Hematol. (2018) 121:90–100. doi: 10.1016/j.critrevonc.2017.11.002, PMID: 29198853

[14] Fuller JT Hartland MC Maloney LT Davison K. Therapeutic effects of aerobic and resistance exercises for cancer survivors: a systematic review of meta-analyses of clinical trials. Br J Sports Med. (2018) 52:1311. doi: 10.1136/bjsports-2017-098285, PMID: 29549149

[15] Campbell KL Winters-Stone KM Wiskemann J May AM Schwartz AL Courneya KS. Exercise guidelines for cancer survivors: consensus statement from international multidisciplinary roundtable. Med Sci Sports Exerc. (2019) 51:2375–90. doi: 10.1249/MSS.0000000000002116, PMID: 31626055 PMC8576825

[16] Ligibel JA Bohlke K May AM Clinton SK Demark-Wahnefried W Gilchrist SC. Exercise, diet, and weight management during cancer treatment: ASCO guideline. J Clin Oncol. (2022) 40:2491–507. doi: 10.1200/JCO.22.00687 35576506

[17] Lin WL Wang RH Chou FH Feng IJ Fang CJ Wang HH. The effects of exercise on chemotherapy-induced peripheral neuropathy symptoms in cancer patients: a systematic review and meta-analysis. Support Care Cancer. (2021) 29:5303–11. doi: 10.1007/s00520-021-06082-3, PMID: 33660078

[18] Kanzawa-Lee GA Larson JL Resnicow K Smith EML. Exercise effects on chemotherapy-induced peripheral neuropathy: a comprehensive integrative review. Cancer Nurs. (2020) 43:E172–85. doi: 10.1097/NCC.0000000000000801 32187026

[19] Lopez-Garzon M Cantarero-Villanueva I Postigo-Martin P González-Santos Á Lozano-Lozano M Galiano-Castillo N. Can physical exercise prevent chemotherapy-induced peripheral neuropathy in patients with cancer? A systematic review and meta-analysis. Arch Phys Med Rehabil. (2022) 103:2197–208. doi: 10.1016/j.apmr.2022.02.008, PMID: 35271844

[20] Molassiotis A Cheng HL Lopez V Au JSK Chan A Bandla A. Are we mis-estimating chemotherapy-induced peripheral neuropathy? Analysis of assessment methodologies from a prospective, multinational, longitudinal cohort study of patients receiving neurotoxic chemotherapy. BMC Cancer. (2019) 19:132. doi: 10.1186/s12885-019-5302-4, PMID: 30736741 PMC6368751

[21] Dorsey SG Kleckner IR Barton D Mustian K O’Mara A St Germain D. The national cancer institute clinical trials planning meeting for prevention and treatment of chemotherapy-induced peripheral neuropathy. J Natl Cancer Inst. (2019) 111:531–7. doi: 10.1093/jnci/djz011, PMID: 30715378 PMC7962883

[22] Page MJ McKenzie JE Bossuyt PM Boutron I Hoffmann TC Mulrow CD. The PRISMA 2020 statement: an updated guideline for reporting systematic reviews. BMJ. (2021) 372:n71. doi: 10.1136/bmj.n71, PMID: 33782057 PMC8005924

[23] Zimmer P Trebing S Timmers-Trebing U Schenk A Paust R Bloch W. Eight-week, multimodal exercise counteracts a progress of chemotherapy-induced peripheral neuropathy and improves balance and strength in metastasized colorectal cancer patients: a randomized controlled trial. Support Care Cancer. (2018) 26:615–24. doi: 10.1007/s00520-017-3875-5, PMID: 28963591

[24] Dhawan S Andrews R Kumar L Wadhwa S Shukla G. A randomized controlled trial to assess the effectiveness of muscle strengthening and balancing exercises on chemotherapy-induced peripheral neuropathic pain and quality of life among cancer patients. Cancer Nurs. (2020) 43:269–80. doi: 10.1097/NCC.0000000000000693, PMID: 30888982

[25] Kleckner IR Park SB Streckmann F Wiskemann J Hardy S Mohile N. Systematic review of exercise for prevention and management of chemotherapy-induced peripheral neuropathy In: Lustberg M Loprinzi C, editors. Diagnosis, management and emerging strategies for chemotherapy-induced neuropathy: A MASCC book. Cham: Springer International Publishing (2021). 183–241.

[26] Higgins JPT Thomas J Chandler J Cumpston M Li T Page MJ.(eds.) Cochrane handbook for systematic reviews of interventions. 2. Chichester: John Wiley & Sons (2019). doi: 10.1002/9781119536604 PMC10284251 31643080

[27] McGuinness LA Higgins JPT. Risk of bias VISualization (robvis): an R package and shiny web app for visualizing risk of bias assessments. Res Synth Methods. (2021) 12:55–61. doi: 10.1002/jrsm.1411 32336025

[28] Béliveau A Boyne DJ Slater J Brenner D Arora P. BUGSnet: an R package to facilitate the conduct and reporting of Bayesian network meta-analyses. BMC Med Res Methodol. (2019) 19:196. doi: 10.1186/s12874-019-0829-2 31640567 PMC6805536

[29] Müller J Weiler M Schneeweiss A Haag GM Steindorf K Wick W. Preventive effect of sensorimotor exercise and resistance training on chemotherapy-induced peripheral neuropathy: a randomised-controlled trial. Br J Cancer. (2021) 125:955–65. doi: 10.1038/s41416-021-01471-1, PMID: 34226683 PMC8476560

[30] Şimşek NY Demir A. Cold application and exercise on development of peripheral neuropathy during taxane chemotherapy in breast cancer patients: a randomized controlled trial. Asia Pac J Oncol Nurs. (2021) 8:255–68. doi: 10.4103/apjon.apjon-2075, PMID: 33850959 PMC8030600

[31] Saraboon C Siriphorn A. Effects of foam pad balance exercises on cancer patients undergoing chemotherapy: a randomized control trial. J Bodyw Mov Ther. (2021) 28:164–71. doi: 10.1016/j.jbmt.2021.07.013, PMID: 34776136

[32] Kleckner IR Kamen C Gewandter JS Mohile NA Heckler CE Culakova E. Effects of exercise during chemotherapy on chemotherapy-induced peripheral neuropathy: a multicenter, randomized controlled trial. Support Care Cancer. (2018) 26:1019–28. doi: 10.1007/s00520-017-4013-0, PMID: 29243164 PMC5823751

[33] Streckmann F Kneis S Leifert JA Baumann FT Kleber M Ihorst G. Exercise program improves therapy-related side-effects and quality of life in lymphoma patients undergoing therapy. Ann Oncol. (2014) 25:493–9. doi: 10.1093/annonc/mdt568, PMID: 24478323

[34] Schwenk M Grewal GS Holloway D Muchna A Garland L Najafi B. Interactive sensor-based balance training in older cancer patients with chemotherapy-induced peripheral neuropathy: a randomized controlled trial. Gerontology. (2016) 62:553–63. doi: 10.1159/000442253, PMID: 26678611 PMC6644035

[35] Vollmers PL Mundhenke C Maass N Bauerschlag D Kratzenstein S Röcken C. Evaluation of the effects of sensorimotor exercise on physical and psychological parameters in breast cancer patients undergoing neurotoxic chemotherapy. J Cancer Res Clin Oncol. (2018) 144:1785–92. doi: 10.1007/s00432-018-2686-5 29943097 PMC11813415

[36] Stuecher K Bolling C Vogt L Niederer D Schmidt K Dignaß A. Exercise improves functional capacity and lean body mass in patients with gastrointestinal cancer during chemotherapy: a single-blind RCT. Support Care Cancer. (2019) 27:2159–69. doi: 10.1007/s00520-018-4478-5, PMID: 30288602

[37] Clark PG Cortese-Jimenez G Cohen E. Effects of reiki, yoga, or meditation on the physical and psychological symptoms of chemotherapy-induced peripheral neuropathy: a randomized pilot study. J Evid-Based Complement Altern Med. (2012) 17:161–71. doi: 10.1177/2156587212450175

[38] Streckmann F Lehmann HC Balke M Schenk A Oberste M Heller A. Sensorimotor training and whole-body vibration training have the potential to reduce motor and sensory symptoms of chemotherapy-induced peripheral neuropathy-a randomized controlled pilot trial. Support Care Cancer. (2019) 27:2471–8. doi: 10.1007/s00520-018-4531-4, PMID: 30382392

[39] Aaronson NK Ahmedzai S Bergman B Bullinger M Cull A Duez NJ. The European Organization for Research and Treatment of Cancer QLQ-C30: a quality-of-life instrument for use in international clinical trials in oncology. J Natl Cancer Inst. (1993) 85:365–76. doi: 10.1093/jnci/85.5.365, PMID: 8433390

[40] Cella D Peterman A Hudgens S Webster K Socinski MA. Measuring the side effects of taxane therapy in oncology: the functional assessment of cancer therapy-taxane (FACT-taxane). Cancer. (2003) 98:822–31. doi: 10.1002/cncr.11578 12910528

[41] Cella DF Tulsky DS Gray G Sarafian B Linn E Bonomi A. The functional assessment of cancer therapy scale: development and validation of the general measure. J Clin Oncol. (1993) 11:570–9. doi: 10.1200/JCO.1993.11.3.570 8445433

[42] Calhoun EA Welshman EE Chang CH Lurain JR Fishman DA Hunt TL. Psychometric evaluation of the Functional Assessment of Cancer Therapy/Gynecologic Oncology Group-Neurotoxicity (Fact/GOG-Ntx) questionnaire for patients receiving systemic chemotherapy. Int J Gynecol Cancer. (2003) 13:741–8. doi: 10.1111/j.1525-1438.2003.13603.x, PMID: 14675309

[43] Tanay MAL Armes J Moss-Morris R Rafferty AM Robert G. A systematic review of behavioural and exercise interventions for the prevention and management of chemotherapy-induced peripheral neuropathy symptoms. J Cancer Surviv. (2023) 17:254–77. doi: 10.1007/s11764-021-00997-w 33710510 PMC9971149

[44] Nuñez de Arenas-Arroyo S Cavero-Redondo I Torres-Costoso A Reina-Gutiérrez S Lorenzo-García P Martínez-Vizcaíno V. Effects of exercise interventions to reduce chemotherapy-induced peripheral neuropathy severity: a meta-analysis. Scand J Med Sci Sports. (2023) 33:1040–53. doi: 10.1111/sms.14360 36972017

[45] Courneya KS McKenzie DC Mackey JR Gelmon K Friedenreich CM Yasui Y. Effects of exercise dose and type during breast cancer chemotherapy: multicenter randomized trial. J Natl Cancer Inst. (2013) 105:1821–32. doi: 10.1093/jnci/djt297, PMID: 24151326

[46] Dannecker EA Koltyn KF. Pain during and within hours after exercise in healthy adults. Sports Med. (2014) 44:921–42. doi: 10.1007/s40279-014-0172-z 24668291

[47] Chou YJ Lai YH Lin BR Liang JT Shun SC. Factors influencing amount of weekly exercise time in colorectal cancer survivors. Cancer Nurs. (2017) 40:201–8. doi: 10.1097/NCC.0000000000000383, PMID: 27135754

[48] Lindell M Grimby-Ekman A. Stress, non-restorative sleep, and physical inactivity as risk factors for chronic pain in young adults: a cohort study. PLoS One. (2022) 17:e0262601. doi: 10.1371/journal.pone.0262601, PMID: 35061825 PMC8782303

[49] Winters-Stone KM Horak F Dieckmann NF Luoh S-W Eckstrom E Stoyles SA. GET FIT: a randomized clinical trial of Tai Ji Quan versus strength training for fall prevention after chemotherapy in older, postmenopausal women cancer survivors. J Clin Oncol. (2023) 41:3384–96. doi: 10.1200/jco.22.01519, PMID: 36888933 PMC10414741

[50] Zhang X Wang A Wang M Li G Wei Q. Non-pharmacological therapy for chemotherapy-induced peripheral neurotoxicity: a network meta-analysis of randomized controlled trials. BMC Neurol. (2023) 23:433. doi: 10.1186/s12883-023-03485-z, PMID: 38082216 PMC10712106

[51] Lehmann HC Wunderlich G Fink GR Sommer C. Diagnosis of peripheral neuropathy. Neurol Res Pract. (2020) 2:20. doi: 10.1186/s42466-020-00064-2, PMID: 33324924 PMC7650053

[52] Timmins HC Li T Kiernan MC Horvath LG Goldstein D Park SB. Quantification of small fiber neuropathy in chemotherapy-treated patients. J Pain. (2020) 21:44–58. doi: 10.1016/j.jpain.2019.06.011, PMID: 31325646

[53] Meregalli C Monza L Jongen JLM. A mechanistic understanding of the relationship between skin innervation and chemotherapy-induced neuropathic pain. Front Pain Res. (2022) 3:1066069. doi: 10.3389/fpain.2022.1066069, PMID: 36582196 PMC9792502

[54] Terkelsen AJ Karlsson P Lauria G Freeman R Finnerup NB Jensen TS. The diagnostic challenge of small fibre neuropathy: clinical presentations, evaluations, and causes. Lancet Neurol. (2017) 16:934–44. doi: 10.1016/s1474-4422(17)30329-0, PMID: 29029847

[55] Chiaramonte R Romano M Vecchio M. A systematic review of the diagnostic methods of small fiber neuropathies in rehabilitation. Diagnostics. (2020) 10:613. doi: 10.3390/diagnostics10090613, PMID: 32825514 PMC7554909

[56] Dobson JL McMillan J Li L. Benefits of exercise intervention in reducing neuropathic pain. Front Cell Neurosci. (2014) 8:102. doi: 10.3389/fncel.2014.00102, PMID: 24772065 PMC3983517

