# Supplementary figures and images for: Exercise intervention for the management of chemotherapy-induced peripheral neuropathy: a systematic review and network meta-analysis
Source: Front Neurol. 2024 Jan 30;15:1346099. doi: 10.3389/fneur.2024.1346099 (PMC10861771; doi:10.3389/fneur.2024.1346099)

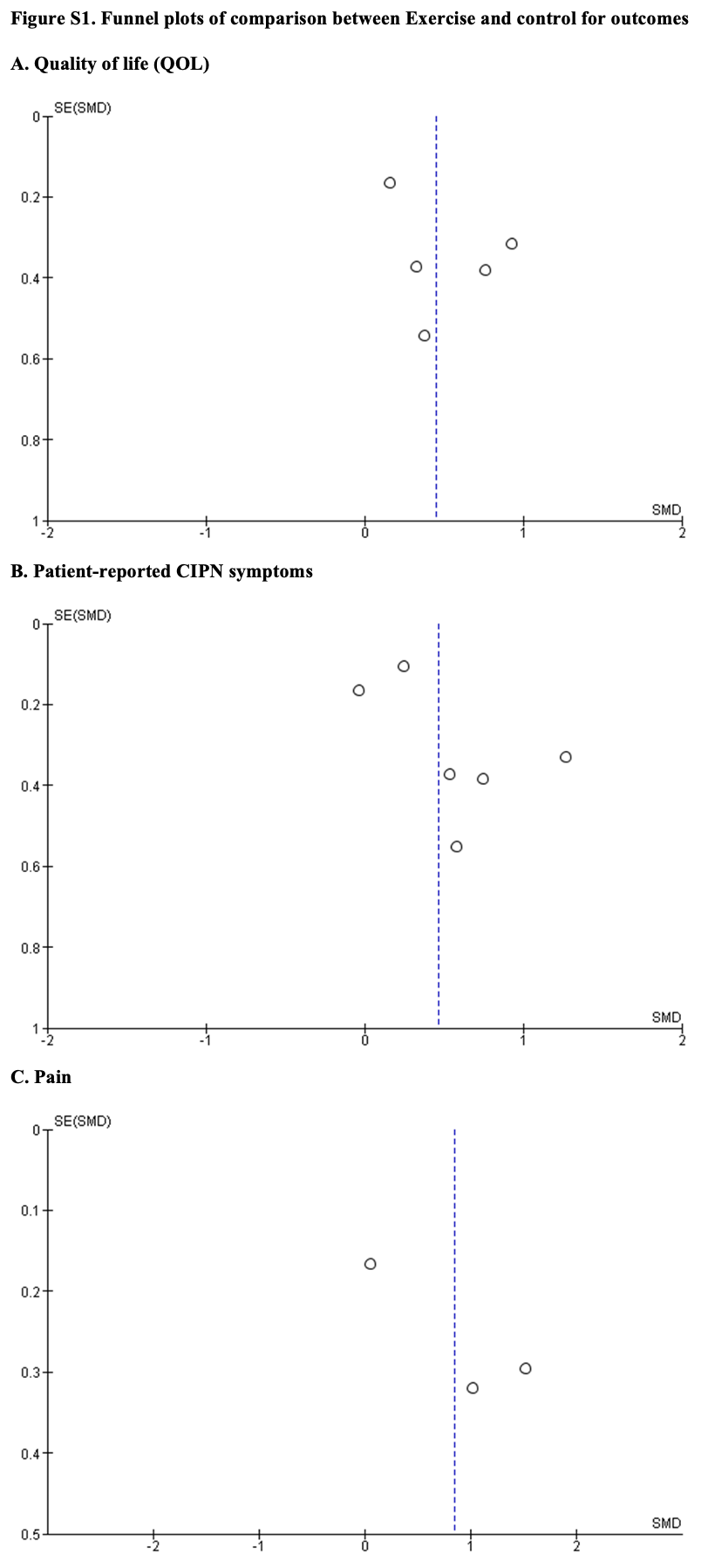

Supplement: Supplementary file 1 [file Image_1.tiff]

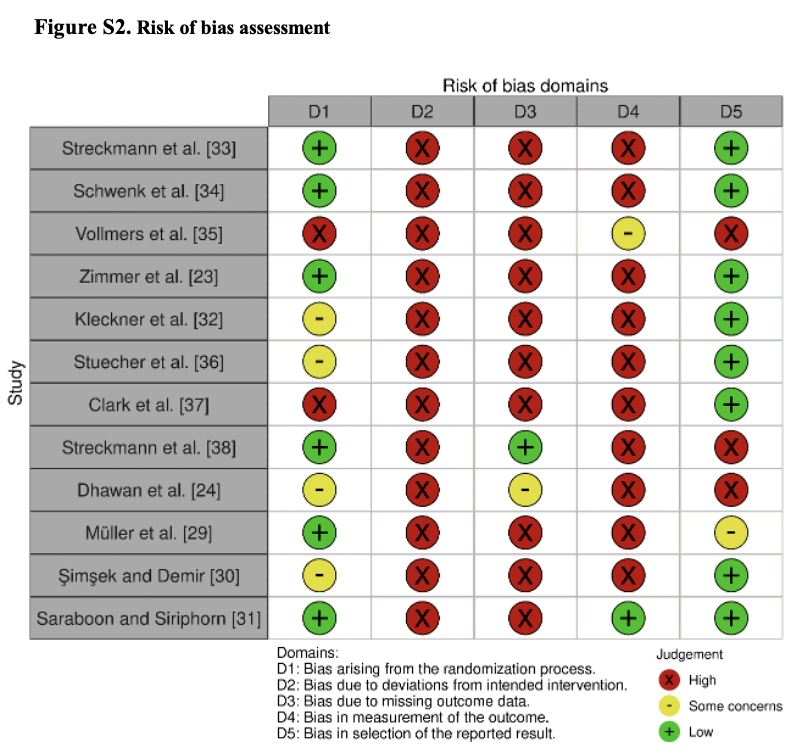

Supplement: Supplementary file 2 [file Image_2.tiff]
