# Supplementary material for: Exercise intervention for the management of chemotherapy-induced peripheral neuropathy: a systematic review and network meta-analysis
Source: Front Neurol. 2024 Jan 30;15:1346099. doi: 10.3389/fneur.2024.1346099 (PMC10861771; doi:10.3389/fneur.2024.1346099)
Supplement: Supplementary file 3 [file Table_1.docx]

Table S1. Overview of included studies

| Author and Year | Exercise Type | Patients | Details of Exercise | Main Outcome Measures † |
| --- | --- | --- | --- | --- |
| Streckmann et al. | Aerobic | Lymphoma, mixed chemotypes | ~1 hourr/day, 2 d/week, 36 weeks | Baseline, after 12, 24, and 36 weeks |
| 2014 [33] | Balance | Unclear baseline CIPN presence | In the clinic, supervised | EORTC QLQ-C30 |
|  | Strength | N = 61 (total)  N = 30 (exercise) | Aerobic: Warm-up on a bicycle dynamometer (60–70% HR max), 10–30 min on a treadmill or bicycle dynamometer (70–80% HR max) at the end of the session | Vibration |
|  |  | Two arms | Balance: Four postural stabilization exercises, progressively increasing exercise difficulty as well as surface instability (20 s exercise +20 s rest for three sets, 1 min rest between different exercises) | Balance control (on static/dynamic surface) |
|  |  |  | Strength: Four different resistance exercises performed with Thera-Bands, at maximum resistance. | Incremental step test |
| Schwenk et al. | Balance | Mixed cancers, chemotypes | 45 min/day, 2 d/week, 4 weeks | Baseline and post-intervention |
| 2016 [34] |  | Established CIPN | In the clinic, unsupervised | FES-1 (fear of falling) |
|  |  | N = 22 (total)  N = 11 (exercise) |  | Balance (feet close/semi tandem, eyes open/feet close, eyes closed) |
|  |  | Two arms |  | Gait-speed variability |
| Vollmers et al. | Balance | Breast cancer, during paclitaxel treatment | 2day/week, during chemotherapy and for 6 weeks after chemotherapy | Baseline, at the last dose of chemotherapy and the 6-weeks follow-up |
| 2018 [35] | Strength | Unclear baseline CIPN presence | Balance: No dose specified | EORTC QLQ CIPN 20 EORTC QLQ-C30 EORTC-BR23 |
|  |  | N = 43 (total)  N = 21 (exercise) | Strength: Six exercises, two sets of 20 reps, RPE: 13-15 (moderate intensity) | Posturometry sway area Fullerton Advanced Balance Scale scores |
|  |  | Two arms |  | Hand dynamometer Chair rising test |
| Zimmer et al. | Aerobic | Gastrointestinal cancers, oxaliplatin treatment | 60 min/day, 2days/week, 8 weeks | Baseline, postintervention (8 weeks), and12 weeks |
| 2018 [23] | Balance | Established CIPN | In a sports center, supervised | FACT/GOG-TOI FACT/GOG-NTX |
|  | Strength | N = 30 (total)  N = 17 (exercise) | Aerobic: cross-trainer, ergometer, or walking; 10 min, 60-70% HRmax | GGT-Reha |
|  |  | Two arms | Balance: 10 min | Strength: e1RM 6MWT |
|  |  |  | Strength: Five exercises; 20 min; 60-80% of e1RM; Borg CR10 scale level: 6 |  |
| Kleckner et al. | Aerobic | Mixed cancers, chemotypes | Daily, 6weeks | Baseline and post-intervention |
| 2018 [32] | Strength | Most patients reported mild baseline CIPN | Home-based, unsupervised | Numbness (NRS) Tingling (NRS) Hot/coldness in hands/feet (NRS) |
|  |  | N = 456 (total)  N = 231 (exercise) | Aerobic: Walking; 60-85% of heart rate reserve |  |
|  |  | Two arms | Strength: Resistance exercise; 3-5 RPE |  |
| Stuecher et al. | Aerobic | Gastrointestinal cancers, mixed chemotypes | 12 weeks | Before chemotherapy, after two cycles, after 12 weeks |
| 2019 [36] |  | Unclear baseline CIPN presence | Home-based, unsupervised | Vibration |
|  |  | N = 44 (total)  N = 22 (exercise) | 150 min, moderate walking per week | Postural sway |
|  |  | Two arms | Borg’s self-rating of RPE of 11-13 | SPPB Gait speed Lower-extremity muscle strength |
| Clark et al. | Yoga* | Mixed cancers, platinum treatment | 60 min/week, 6 weeks | Baseline and post-intervention |
| 2012 [37] |  | Established CIPN | In the clinic, supervised | FACT-GOG-Ntx Brief Symptom Inventory – 18 |
|  |  | N = 36 (total)  N = 9 (exercise) | Low intensity |  |
|  |  | Four arms |  |  |
| Streckmann et al. | Balance | Mixed cancers, chemotypes | 2days/week, 6 weeks | Baseline and post-intervention |
| 2019 [38] |  | Established CIPN | In the clinic, supervised | FACT GOG-Ntx EORTC-QLQ-C30 Pain-DETECT |
|  |  | N = 40 (total)  N = 20 (exercise) | Balance: On progressively unstable surfaces; Four exercises; Three sets of 20 seconds | Achilles tendons reflex Deep sensitivity Patellar tendon reflex Light-touch perception Sense of position Nerve conduction velocity and amplitude |
|  |  | Four arms | Whole-body vibration: Stand on a vibration platform; four sets of 30 seconds to 1-min | Balance control |
|  |  | Balance/Whole-body vibration/Control/Healthy control |  | Lower-leg strength Gait speed Postural sway |
| Dhawan et al. | Balance | Mixed cancers, carboplatin + paclitaxel | 30 min/day, daily, 10 weeks | Baseline and post-intervention |
| 2020 [24] | Strength | Established CIPN | Home-based, unsupervised | CIPNAT LANSS EORTC-QLQ |
|  |  | N = 45 (total)  N = 22 (exercise) |  |  |
|  |  | Two arms |  |  |
| Müller et al. | Balance | Mixed cancers, chemotypes | During chemotherapy | Baseline, post-intervention, and 3 and 6 weeks after the intervention |
| 2021 [29] | Strength | No baseline CIPN | Balance: 35 min/day, 3 times a week, at home (unsupervised) or in the hospital (supervised) | TNS EORTC QLQ-CIPN 15 EORTC QLQ-C30 FES-1 (fear of falling) |
|  |  | N = 170 (total)  N = 112 (exercise) | Strength: 45 min/day, twice a week, machine-based & 15 min/day, once a week, home-based | Nerve conduction studies |
|  |  | Three arms |  | Postural control (Balance) |
|  |  | Balance/Strength/Control |  | Lower-extremity score |
|  |  |  |  | Chemotherapy completion rate |
| Şimşek and Demir | Balance | Breast cancer, mixed chemotypes | 15-30 min/day, 5d/week, 12 weeks | Baseline and post-intervention |
| 2021 [30] | Strength | Established CIPN | Home-based, supervised | CIPN Assessment Tool |
|  |  | N = 90 (total)  N = 30 (exercise) | Balance: Four exercises |  |
|  |  | Three arms | Strength: Seven exercises |  |
|  |  | Exercise/cold application/control | 10 reps/set for the first 3 weeks, then 20 reps/set for 3 weeks, and then 30 reps/set for 3 weeks |  |
| Saraboon and Siriphorn | Balance | Ovary or cervix cancer, paclitaxel regimens | During chemotherapy | Baseline, during intervention (4 weeks) and post-intervention (6 weeks) |
| 2021 [31] |  | No baseline CIPN | 60 min/day, 2days/week, 6 weeks | FAB |
|  |  | N = 30 (total)  N = 15 (exercise) |  | MDNS |
|  |  | Two arms |  | FACT-Taxane |
|  |  |  |  | SPPB |

†, Primary endpoint is written in bold and underlined. *, Relaxation-based hatha yoga consists of weekly lessons and daily self-practice.

CIPN: chemotherapy-induced peripheral neuropathy, CIPNAT: Chemotherapy-Induced Peripheral Neuropathy Assessment Tool, DASH: Disability of the Arm, Shoulder, and Hand, e1RM: estimated one-repetition maximum, EORTC QLQ: the European Organization for Research and Treatment of Cancer core quality of life questionnaire, FAB: Fullerton Advanced Balance, FACT-GOG: Functional Assessment of Cancer Therapy/Gynecologic Oncology Group, TOI: Trial Outcome Index, Ntx: Neurotoxicity, FACT-Taxane: Functional Assessment of Cancer Therapy-Taxane, FES-1: falls efficacy scale-1, HR: heart rate, LANSS: Leeds Assessment of Neuropathic Symptoms and Sign, MDNS: Michigan Diabetic Neuropathy Score, NPRS: Numeric Pain Rating Scale, NRS: number rated scale, RPE, rate of perceived exertion, S-LANSS: Self-report version of Leeds Assessment for Neuropathic Symptoms and Signs, SPPB: short physical performance battery, TNS: total neuropathy score
