# Supplementary material for: Exercise intervention for the management of chemotherapy-induced peripheral neuropathy: a systematic review and network meta-analysis
Source: Front Neurol. 2024 Jan 30;15:1346099. doi: 10.3389/fneur.2024.1346099 (PMC10861771; doi:10.3389/fneur.2024.1346099)
Supplement: Supplementary file 4 [file Table_2.DOCX]

Table S2. Summary of main results regarding the effect of exercise on outcomes

| Study | Quality of Life (QOL) | Patient-reported CIPN | Pain | Clinical assessments of CIPN signs | Balance measures | Physical functional assessments |
| --- | --- | --- | --- | --- | --- | --- |
| Intergroup comparison | | | | | | |
| Streckmann et al. 2014 [33] | Improvement in QOL within the first 12 weeks  Δ_T1–T0_ (Mean): IG 9.1, CG -6.15, P = 0.028 |  | No significant difference | Reduced peripheral deep sensitivity after 36 weeks.  IG 87.5% (symptom diminished), CG 0%, P < 0.001 | Reduced sway paths after 36 weeks  Δ_T3–T0_: static left P = 0.035, dynamic left P = 0.007, dynamic right P = 0.045  Improvement in failed attempts  Δ_T3–T0_: static left P = 0.024, dynamic left P = 0.014, dynamic right P < 0.001  Improvement in time to regain balance after 36 weeks.  Δ_T3–T0_ (Median): IG -0.26, CG 0.2, P = 0.045 | No significant difference |
| Schwenk et al. 2016 [34] |  | No significant difference |  |  | Reduced sway of hip, ankle, and center of mass (CoM) post intervention  Feet close – eyes open P = 0.010–0.022 (except anterior-posterior CoM sway)  Semitandem – eyes open  P = 0.008–0.035 (except ankle sway) | No significant difference |
| Vollmers et al. 2018 [35] | No significant difference |  |  |  | Smaller sway area  Monopedal stance  T1 (after the last dose) (left and right) both P < 0.001, T2 (6 weeks follow-up) (left) P = 0.003, T2 (right) P<0.01  Bipedal stance  P = 0.039  Improvement in postural stability  Δ_T1-T0_: IG +1.35, CG -2.84, P < 0.001 | Less loss of strength in hand dynamometry  IG 0.60, CG -1.60, P = 0.029 |
| Zimmer et al. 2018 [23] | No significant difference | Less aggravation of neuropathic symptoms  Mean (T0/T1[after the intervention]/T2[after 4 weeks follow-up]): IG 33.12/35.24/34,  CG 34.08/28.97/29.43  Δ_T1–T0_: P = 0.002, Δ_T2–T0_: P = 0.015 |  |  | Improvement in advanced static balance  Mean (T0/T1/T2):  IG 11.47/13.35/12.47,  CG 11/9.92/9.62  Δ_T1–T0_: P = 0.025, Δ_T2–T0_: P = 0.025 | Increased muscle strength  Bench press  Δ_T1–T0_: P = 0.014, Δ_T2–T0_: P = 0.014  Leg press  Δ_T1–T0_: P = 0.001, Δ_T2–T0_: P = 0.011  Lat pulldown  Δ_T1–T0_: P = 0.022, Δ_T2–T0_: P = 0.031 |
| Kleckner et al. 2018 [32] |  | Less severe CIPN symptoms at postintervention  Hot/coldness in hands/feet  Coefficient: -0.46, P = 0.045 |  |  |  |  |
| Stuecher et al. 2018 [36] |  |  |  |  | Improvement in postural sway after 12 weeks  Δ_T2–T0_ (Mean): IG -58.8, CG 58.7, P = 0.003 (Group effect) | No significant difference |
| Streckmann et al. 2019 [38] | No significant difference | No significant difference | Reduction of pain  F(2,25) = 3.575, P = 0.043 | Improvement in Achilles tendon reflex  F(2,26) = 4.791, P = 0.017  Improvement in patellar tendon reflex  F(2,25) = 4.564, P = 0.020  Improvement in peripheral deep sensitivity  F(2,25) = 5.548, P = 0.010 |  |  |
| Dhawan et al. 2020 [24] | Better QOL after 10 weeks  Mean: IG 61.7, CG 43.1, P = 0.002 | Less severe, distressing, or frequent CIPN symptoms after 10 weeks  Mean: IG 83.1, CG 140.8, P < 0.0001 | Less neuropathic pain after 10 weeks  Mean: IG 10.7, CG 15.8, P = 0.001 |  |  |  |
| Müller et al. 2021 [29] | No significant difference | No significant difference | No significant difference | No significant difference | Keeping average time of standing on one leg with open eyes at 3 weeks after chemotherapy  Adjusted between-group difference:  Sensorimotor training vs. CG 2.2, P = 0.045  Resistance training vs. CG 2.3, P = 0.023 | Maintained muscle strength  Adjusted between-group difference:  Resistance training vs. CG 11.1, P = 0.045 |
| Şimşek and Demir 2021 [30] |  | Less severe CIPN symptoms after 12 weeks  Numbness in the hand  Mean: IG 1.9, Cold application 3.0, CG 3.1, P = 0.009  Numbness in the foot  Mean: IG 3.8, Cold application 4.0, CG 5.5, P = 0.009 | No significant difference |  |  |  |
| Saraboon and Siriphorn  2021 [31] | No significant difference | No significant difference |  |  | Invariant balance performance  Mean: IG 37.40, CG 34.13, P < 0.01 | Maintained baseline physical performance  Mean: IG 11.47, CG 10.67, P = 0.03 |
| Intragroup comparison | | | | | | |
| Clark et al. 2012 [37] | No significant difference | There were no significant changes in IG whereas neuropathic symptoms worsened in CG.  Mean, pre vs. post  IG: 30.31 vs. 32.43, P = 0.278  CG: 31.14 vs. 27.86, P = 0.03 |  |  |  |  |

CIPN: chemotherapy-induced peripheral neuropathy, IG: intervention group, CG: control group
