# Supplementary material for: Exercise intervention for the management of chemotherapy-induced peripheral neuropathy: a systematic review and network meta-analysis
Source: Front Neurol. 2024 Jan 30;15:1346099. doi: 10.3389/fneur.2024.1346099 (PMC10861771; doi:10.3389/fneur.2024.1346099)
Supplement: Supplementary file 5 [file Table_3.DOCX]

Table S3. The detail of the search strategy in this study

| #1 | "survivor" or "survivor AND cancer" or "cancer survivor" or "cancer" |
| --- | --- |
| #2 | neuropathy or "neuropathies" or "chemotherapy-induced" or "chemotherapy-induced neuropathy" or "chemotherapy-induced peripheral neuropathy" or "CIPN" or "peripheral nervous system/drug effects" or "peripheral nerve diseases/chemically induced" or "antineoplastic agents/adverse effects" or "neoplasms/drug therapy" or "neoplasms/complications" |
| #3 | #1 AND #2 |
| #4 | "motor activity"[mesh] or "exercise"[mesh] or "exercise therapy"[mesh] or "exercise"[tiab] or "exercises"[tiab] or "training"[tiab] |
| #5 | #3 AND #4 |
| #6 | animal[mh] NOT (animal[mh] AND humans[mh]) |
| #7 | #5 NOT #6 |
