# Supplementary material for: Exercise intervention for the management of chemotherapy-induced peripheral neuropathy: a systematic review and network meta-analysis
Source: Front Neurol. 2024 Jan 30;15:1346099. doi: 10.3389/fneur.2024.1346099 (PMC10861771; doi:10.3389/fneur.2024.1346099)
Supplement: Supplementary file 6 [file Table_4.docx]

Table S4. Enrollment, completion rate and adherence

| Author and Year | Adverse events | Enrollment rate | Completion rate | Adherence |
| --- | --- | --- | --- | --- |
| Streckmann et al. 2014 [33] | No AEs occurred. | ND | 91% | mean: 65% |
| Schwenk et al. 2016 [34] | No AEs occurred. | 85% | 86% | ND |
| Vollmers et al. 2018 [35] | Based on the questionaries, there were few differences between intervention and control group. | 40% | 84% | ND |
| Zimmer et al. 2018 [23] | No serious AEs were observed. | ND | 80% | mean: 88% |
| Kleckner et al. 2018 [32] | 5 grade-3–5 AEs occurred. All were unrelated to the exercise intervention. | ND | 85% | Patients in intervention group walked 0.27 miles per day more than patients in control group. |
| Stuecher et al. 2019 [36] | No AEs occurred. | ND | 97% | 98% |
| Clark et al. 2012 [37] | ND | 100% | 72% | ND |
| Streckmann et al. 2019 [38] | No AEs occurred. | ND | 64% | mean: 81% |
| Dhawan et al. 2020 [24] | No exercise-related AEs were observed. | 28% | 91% | 68% |
| Saraboon and Siriphorn,021 [31] | ND | 83% | 100% | 87% |
| Müller et al. 2021 [29] | Mild training-associated AEs without indication for medical treatment occurred in 21% in the SMT group and 25% in the RT group. | 25% | 84% | 55% in the SMT group 49% in the RT group |
| Şimşek and Demir2021 [30] | ND | 71% | 100% | ND |

AE: adverse event, ND: not described, RT: resistance training, SMT: sensorimotor training
